# *Dendrobium officinale* Polysaccharide Prevents Diabetes via the Regulation of Gut Microbiota in Prediabetic Mice

**DOI:** 10.3390/foods12122310

**Published:** 2023-06-08

**Authors:** Haodong Liu, Yan Xing, Yinbo Wang, Xinxiu Ren, Danyang Zhang, Jianying Dai, Zhilong Xiu, Shiqiang Yu, Yuesheng Dong

**Affiliations:** 1 School of Bioengineering, Dalian University of Technology, Dalian 116024, China; liu18742060205@163.com (H.L.); docxyxy@163.com (Y.X.); renxinxiu@mail.dlut.edu.cn (X.R.); zhangdanyang96@mail.dlut.edu.cn (D.Z.); jydai@dlut.edu.cn (J.D.); zhlxiu@dlut.edu.cn (Z.X.); 2 Dianxi Research Institute, Dalian University of Technology, Baoshan 678000, China; w18287529596@126.com (Y.W.); shiqiang.yu@hotmail.com (S.Y.)

**Keywords:** *Dendrobium officinale* polysaccharide, prediabetes, insulin resistance, gut microbiota, intestinal inflammation, short-chain fatty acids

## Abstract

*Dendrobium officinale* polysaccharide (DOP), which serves as a prebiotic, exhibits a variety of biological activities, including hypoglycemic activities. However, the effects of DOP on diabetes prevention and its hypoglycemic mechanisms are still unclear. In this study, the effects of DOP treatment on the prediabetic mice model were studied and the mechanism was investigated. The results showed that 200 mg/kg/d of DOP reduced the relative risk of type 2 diabetes mellitus (T2DM) from prediabetes by 63.7%. Meanwhile, DOP decreased the level of LPS and inhibited the expression of TLR4 by regulating the composition of the gut microbiota, consequently relieving the inflammation and alleviating insulin resistance. In addition, DOP increased the abundance of SCFA (short chain fatty acid)-producing bacteria in the intestine, increased the levels of intestinal SCFAs, promoted the expression of short-chain fatty acid receptors FFAR2/FFAR3, and increased the secretion of the intestinal hormones GLP-1 and PYY, which helped to repair islet damage, suppress appetite, and improve insulin resistance. Our results suggested that DOP is a promising functional food supplement for the prevention of T2DM.

## 1. Introduction

Prediabetes is the initial period of type 2 diabetes mellitus (T2DM) in which blood sugar levels are higher than normal but not sufficient to meet the criteria of T2DM. It is characterized by impaired fasting glycemia (IFG) and impaired glucose tolerance (IGT) [1,2]. The World Health Organization (WHO) and the American Diabetes Association (ADA) define prediabetes as postprandial blood glucose (PBG) levels that range from 7.8 to 11.0 mM (IGT) and fasting blood glucose (FBG) levels that range from 5.6 to 6.9 mM (IFG) [2,3]. The prevalence of prediabetes is increasing globally, with experts predicting that over 470 million people will have prediabetes by 2030. Approximately 5–10% of people with prediabetes become diabetic each year [2]. The prevalence of diabetes is increasing annually, which is associated with the enormous number of prediabetes patients. The development of diabetes from prediabetes can be prevented with proper lifestyle changes or drug treatment. Therefore, the intervention of prediabetes is the final opportunity to prevent T2DM, which has great economic and social significance.

At present, clinical trials of several antidiabetic drugs had been conducted to treat prediabetes, including acarbose, metformin, troglitazone exenatide, and liraglutide. However, due to the unsatisfactory efficacy of the treatment and side effects, for example, the gastrointestinal side effects for the acarbose, metformin, and liraglutide, and hepatotoxic for the troglitazone [4,5,6,7], none of them had been launched to the market [2]. However, the active ingredients in natural plants have the advantages of mild efficacy, low side effects, and multi-target intervention. Previous studies in our laboratory have found that some flavonoids and alkaloids, for instance, baicalein and oroxin A from *Oroxylum indicum*, and DNJ from *Morus alba* L., reduced blood glucose levels and improved insulin sensitivity in prediabetic mice, significantly reducing the risk of progression to T2DM in prediabetic mice [8,9,10]. However, the effects of polysaccharides from natural products on prediabetes treatment were not well documented.

*Dendrobium officinale* Kimura et Migos is the perennial herb of the genus *Dendrobium* in the Orchidaceae family. *D. officinale* is mainly grown in the Southwest of the country in areas, such as Guangxi, Guizhou, Zhejiang, Yunnan, and Anhui, in China. *D. officinale* showed a variety of functions, such as antioxidation, immunomodulation, and anti-cancer [11]. The safety of *D. officinale* has been widely demonstrated, and *D. officinale* has been listed as a “medicinal and edible plant” by the Chinese government [12]. *D. officinale* is widely used to prepare beverages, porridges, and soups to cure constipation in China and Southeast Asia, as well as in new sources of specialty cosmetic materials, on account of its highly edible and medicinal values [13]. The main components in *D. officinale* are polysaccharides, alkaloids, and phenolic compounds. Among them, the *D. officinale* polysaccharide (DOP) was shown to be the major active component, and the content of DOP in the dried stems of *D. officinale* ranges from 10% to 50% [14,15,16]. Previous studies reported that the molecular weights of DOPs ranged from 2.53 to 1930 kDa [16] and the monosaccharide composition was mainly glucose (Glu) and mannose (Man); thus, DOP was usually considered a kind of glucomannan. Most DOPs have a backbone consisting of 1,4-β-D-Manp and 1,4-β-D-Glcp, with acetyl groups in varying amounts and positions with or without branches [16]. Many studies reported the benefits of glucomannans on health; for example, the konjac glucomannans showed anti-diabetes, as well as laxative and anti-inflammatory activities [17]. The effects of DOP on the treatment of diabetes and other metabolic diseases were also reported. In in vivo studies, DOP showed a significant hypoglycemic effect [18] and improvement in symptoms including oxidative stress, inflammation, and hepatic lipid accumulation in the liver [19]. DOPs of different molecular weights were also shown to alleviate abnormal glucolipid metabolism, organ dysfunction, and gut microbiota dysregulation in T2DM mice [20]. However, whether DOP has the beneficial effect of reducing the progress from prediabetes to T2DM still needs to be investigated.

Some studies also demonstrated the mechanism of DOP in T2DM treatment. The hypoglycemic effect of DOP was attributed to the regulation of glycogen synthesis and glucose-metabolizing enzyme activity through the activation of the PI3K/Akt signaling pathway [18], the promotion of hepatic glycogen synthesis, and the inhibition of hepatic glycogen degradation through inhibition of the glucagon-mediated cAMP–PKA signaling pathway [21]. Numerous types of research have demonstrated a tight link between gut microbiota and T2DM [22,23,24]. As DOP was not easily digested and absorbed after entering the digestive tract, most DOP entered the colon and was decomposed by the gut microbiota [25]. Therefore, regulating the gut microbiota was suggested as one of the major mechanisms of DOP in T2DM treatment. Previous research also linked the hypoglycemic effect of DOP with the improvement of SCFA levels produced by changes in the composition and abundance of intestinal bacteria [20]. However, to the best of our knowledge, the exact mechanism that dictates how DOP affects glycemic levels and insulin resistance through gut microbiota regulation remains unclear. This mechanism will provide new insights for DOP to prevent the progression from prediabetes to T2DM.

In this study, the administration effect of DOP on a high-fat and high-sugar diet (HFHSD) and streptozotocin (STZ)-induced prediabetic mice was evaluated. In addition, the mechanism of DOP exerting the regulation of glucose metabolism and ameliorating insulin resistance through gut microbiota was investigated.

## 2. Materials and Methods

### 2.1. Materials and Reagents

*D. officinale* was collected from Libo County (Guizhou, China) and authenticated by Prof. Baolin Guo, the Institute of Medicinal Plant Development (IMPLAD), Peking Union Medical College. C57BL/6 mice were purchased from Changsheng Biotechnology Co., Ltd. (Benxi, China). The standard maintenance diet and high-fat high-sugar diet (HFHSD) were purchased from SYSE Bio-tech Co., Ltd. (Changzhou, China). Streptozotocin was purchased from Sigma Co. (Saint Louis, MO, USA). The insulin detection kit, Mouse Peptide YY (PYY), GLP-1, LPS, TNF-α, and IL-6 ELISA Kits were purchased from Shanghai Enzyme-linked Biotechnology (Shanghai, China). Assay kits for triglyceride (TG), total cholesterol (T-CHO), alanine aminotransferase (ALT), and aspartate aminotransferase (AST) were purchased from Jiancheng Bioengineering Institute (Nanjing, China). The primers for FFAR2, FFAR3 (Table 1), and β-actin were purchased from Sangon Biotech Co., Ltd. (Shanghai, China). The reagents used for RT-PCR were from TaKaRa PrimeScript^TM^RT reagent Kit with gDNA Eraser (Perfect Real Time) and TaKaRa Green^®^ Premix Ex Taq^TM^ II (Til RnaseH Plus). The total protein extraction kit and BCA protein assay kit were purchased from Solarbio Science & Technology Co., Ltd. (Beijing, China). The antibodies for TLR4, IKKα, p-IκBα, IκBα, NF-κB p65, β-actin, and horseradish peroxidase-conjugated goat-antirabbit IgG were purchased from Beyotime Biotechnology Co., Ltd. (Shanghai, China). Other chemicals were supplied by Damao Co., Ltd. (Tianjin, China).

### 2.2. Preparation of DOP

*D. officinale* powder was soaked in 95% ethanol overnight. After soaking, *D. officinale* powder was filtered and dried to completely evaporate the residual ethanol in it. After that, the crude polysaccharide was extracted by the water extraction and alcohol precipitation method. The crude polysaccharide was dissolved with the appropriate amount of water, and the protein was removed by the sevag method until no precipitation was precipitated from the middle phase. The upper phase was decolorized by adding activated carbon and filtered, after which the filtrate was dialyzed in running water for 12 h and pure water for 12 h. The solution in the dialysis bags was then collected, precipitated overnight by 75% final concentration ethanol in the refrigerator at 4 °C, and lyophilized to obtain *D. officinale* polysaccharide (DOP).

### 2.3. Purity Determination of DOP

A total of 10 mg of DOP was weighed and dissolved completely with 1 mL of pure water as the sample solution to be tested. The concentration of polysaccharide (x) in the sample solution was determined by the phenol-sulphuric acid method, which was repeated three times. The purity of the DOP (ω) is calculated by Equation (1).

ω = (x/10) × 100%(1)

### 2.4. Analysis of Monosaccharide Composition

The monosaccharide composition determination was carried out using the PMP (5-Methyl-2-phenyl-1,2-dihydropyrazol-3-one)-HPLC-PDA method with modifications from the literature [15,26]. A 2 mg polysaccharide sample was weighed and dissolved in 2 mL 2 M TFA solution and acidified in an oil bath at 120 °C for 4 h. After the acid hydrolysis and cooling, methanol was added and rotary evaporated several times to remove unreacted TFA. A total of 0.2 mL hydrolysate was added to a 0.4 mL 0.3 M NaOH solution and a 0.4 mL 0.5 M PMP methanol solution, and the derivatization was carried out in the water bath at 70 °C for 60 min. After cooling, the 0.4 mL 0.3 M HCl solution was added to neutralize the alkali, and 1 mL chloroform was added to extract the upper layer three times. The 1 mg/mL monosaccharide standard was configured with the same derivatization method as the polysaccharide sample for HPLC analysis. Chromatographic condition: the Waters system was equipped with an Agilent ZORBAX SB C18 column (4.6 × 250 mm, 0.5 μm). Mobile phase: Acetonitrile: 0.02 M ammonium acetate solution = 20:80, the flow rate was 1 mL/min, and the injection volume was 20 μL.

### 2.5. Animals and Experimental Protocols

In the present study, normal mice (male, body mass 18–22 g, n = 48; male mice were selected for the animal model based on the findings from previous studies that male rodents are more susceptible to the diabetogenic action of STZ than females [27], due to the suggestion that estrogen can reduce the risk of T2DM [28]) were housed in a specific-pathogen-free (SPF) animal room under the controlled temperature of 23 °C and 60% relative humidity and subjected to a 12 h:12 h light–dark cycle (lights on at 8:00). Animals were acclimatized 1 week prior to the experiment and provided with a standard maintenance diet and water.

The procedure of the induction of the prediabetic mice with HFHSD and STZ (50 mg/kg) was applied according to the previous protocol with modifications [8,29]. Oral glucose tolerance test (OGTT) was performed on Days 7, 14, and 21 after the STZ injection. Mice meeting the criteria of the prediabetic model (IFG or IGT) were randomly divided into 3 groups (n = 12, 4 mice in each cage) and given 0.9% NaCl (MD group), metformin (200 mg/kg/d, MET group), or DOP (200 mg/kg/d, DOP group) for 6 weeks and the above grouped mice were fed with HFHSD. Normal mice (n = 12, 4 mice in each cage) were fed with the standard maintenance diet as the control (NC group). Furthermore, these mice were provided with food and water.

During the experimental period, the blood glucose of the mice was measured weekly and assessed to calculate the relative risk reduction of diabetes (RRRD) in the administered group according to Equation (2) [8].

RRRD = (IDC − IDT)/IDC(2)

where IDT was the incidence of diabetes in the treatment (MET or DOP) group and IDC was the incidence of diabetes in the control (MD) group.

DM-FREE (diabetes mellitus-free) = (1 − IDT or IDC) × 100%

At the end of the experiment, the mice were euthanized after anesthesia using 2.5% Avertin. The blood was obtained by cardiac puncture from anesthetized mice and collected in tubes containing 1% heparin as an anticoagulant, and the supernatant was centrifuged to obtain plasma. The tissues were collected and stored at −80 °C. The feces and colon contents were collected under aseptic conditions to analyze the content of short-chain fatty acids (SCFAs) and the composition of the gut microbiota. Part of the fresh tissues was fixed in 4% paraformaldehyde for histology analysis. 

All animal experiments were approved by the Dalian University of Technology Bioethics and Medical Ethics Committee (the approval code is DUTSBE2300414-01) whose permission was in accordance with the National Research Council’s Guide for the care and use of laboratory animals. 

### 2.6. Oral Glucose Tolerance Test (OGTT) and Insulin Tolerance Test (ITT)

The OGTT was performed according to previous literature [9,30]. The mice fasted with free access to water for 14 h, and each mouse was administered 2 g/kg glucose by oral gavage. Blood glucose (BG) levels of mice were measured by tail vein blood sampling using a Roche glucometer at 0 min, 30 min, 60 min, 90 min, and 120 min after glucose oral gavage. The ITT was performed according to previous literature [9]. The mice fasted with free access to water for 5 h, and each mouse was administered 0.4 U/kg insulin by intraperitoneal injection. Blood glucose (BG) levels of mice were measured in the same way as the OGTT after insulin intraperitoneal injection. The area under the plasma glucose curve (AUC) was calculated using Equation (3):

AUC = (BG0 min + 2 × BG30 min + 2 × BG60 min + 2 × BG90 min + BG120 min) × 30 min × 0.5(3)

### 2.7. Determination of Plasma Biomarkers and HOMA-IR

Plasma biomarkers (TG, T-CHO, ALT, AST, PYY, GLP-1, LPS, TNF-α, and IL-6) and fasting insulin levels were determined with the respective assay kit according to the manufacturer’s instructions. The homeostasis model assessment (HOMA)-insulin resistance (HOMA-IR) index was calculated according to the formula reported in the existing literature as Equation (4):

HOMA-IR = fasting insulin level (mIU/mL) × fasting glucose level (mM)/22.5(4)

### 2.8. Histopathological Observation

The tissues fixed with 4% paraformaldehyde were dewaxed with xylene and ethanol gradient. The sections were stained with hematoxylin-eosin (HE), dehydrated with xylene and ethanol gradient, and sealed with neutral gum. The stained sections were observed with an IX83 inverted microscope (Olympus Co., Tokyo, Japan), and the images were recorded and analyzed.

### 2.9. Quantitative Real-Time (RT)-PCR

RNA from tissues was extracted using the Trizol method. The RNA quality and concentration were determined using NanoDrop and were adjusted to 1000 ng/L using RNase-free water. The assay was performed according to the instructions of the kit using β-actin as the internal reference gene. The Applied Biosystems 7500 Real-Time PCR System was used for the detection of each target gene. The data were analyzed using the 2^−ΔΔCT^ method to calculate the relative expression levels of each gene in different groups.

### 2.10. Western Blot Analysis

Total tissue protein was extracted using the total protein extraction kit. About 50 mg of the preserved tissue sample was placed in an EP tube and 1 mL of pre-mixed protein lysate was added. The mixture was then homogenized using the ultrasonic cell grinder on ice. The supernatant was collected by centrifugation at 12,000 rpm for 10 min, and the protein concentration was determined by the BCA method. After determination, the protein samples were added to the loading buffer, placed in the boiling bath for 3–5 min, and then stored in the refrigerator. 

Western blotting was performed according to the procedures previously published [29]. The primary antibodies used in this experiment included TLR4 (1:1000), IKKα (1:1000), p-IκBα (1:1000), NF-κB p65 (1:1000), and β-actin (1:1000).

### 2.11. In Vitro Culture of the Gut Microbiota

The procedure of the culture of the gut microbiota in vitro was based on published literature with minor modifications [31]. Fresh feces from prediabetic mice were collected and fecal homogenates (10%, *w*/*v*) were prepared using 0.1 M PBS. The fecal homogenates (10% inoculum volume) were inoculated into GAM medium under anaerobic conditions and then fermented anaerobically at 37 °C for 12 h. After fermentation, the bacteria were centrifuged, collected, and stored at −80 °C.

### 2.12. Fecal Microbiota 16S rRNA High-Throughput Sequencing

The 16S rRNA sequencing and analysis were performed according to the previous literature. The fecal samples (n = 4) were handed over to Hangzhou Guhe Information Technology Co., Ltd. (Hangzhou, China); then, samples were quality checked, and PCR amplification was carried out after passing the quality check. A region or regions of variation were selected, and general primers were designed for amplification. After amplification, the PCR products were tested by electrophoresis, and the positive and negative controls were normal for the same batch. The libraries that pass the test were then subjected to high-throughput sequencing using the Illumina NovaSeq platform. The sequencing data were used for species identification analysis.

### 2.13. Fecal SCFA Measurement

The quantitative analysis of fecal SCFAs was performed as previously described with slight modifications [29]. About 50 mg of mouse feces was weighed with 1 mL of anhydrous methanol and vortex shook for 10 s. The mixture was then centrifuged at 10,000× *g* for 5 min. The supernatant was analyzed on the Agilent gas chromatography system and used FFAP capillary column (30 m × 0.25 mm × 0.25 μm) with high-purity nitrogen as the carrier gas at a flow rate of 2.96 mL/min, a splitting ratio of 1:10, an injection volume of 1 μL, and a temperature of 250 °C for both the injector and the flame ionization detector (FID). The column ramp-up procedure was as follows: the initial temperature was 80 °C for 2 min, ramped up to 180 °C at a rate of 10 °C/min, and held at 180 °C for 5 min.

### 2.14. Statistical Analysis

Statistical analysis was performed using GraphPad 9.0 and SPSS 23.0 (IBM Corporation) software. Data were expressed as mean ± SEM. Statistical differences in four groups were calculated with a one-way ANOVA followed by Tukey’s post-hoc test (LSR) at a 95% confidence interval, and a *p*-value < 0.05 was considered statistically significant.

## 3. Results

### 3.1. DOP Characterization 

**Figure 1:**
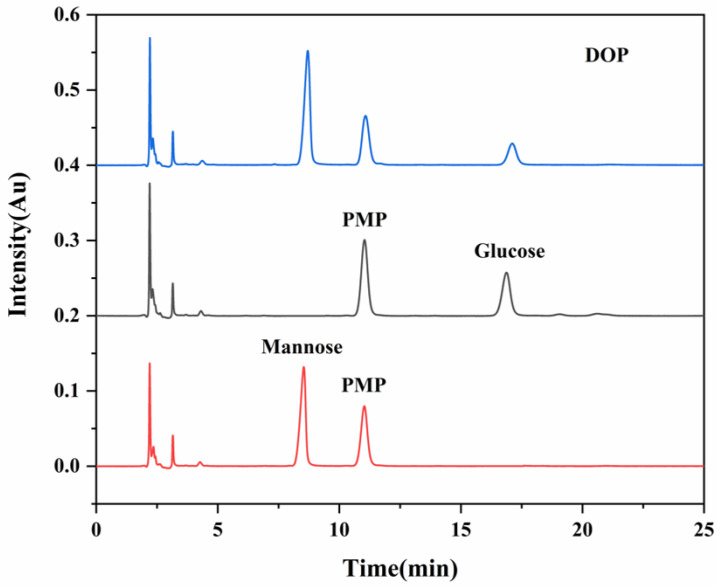
The monosaccharide composition analysis of DOP with PMP-HPLC. The curves represent the PMP-HPLC results of DOP, glucose standard, and mannose standard from top to bottom, respectively.

After hot water extraction and alcohol precipitation, crude DOP was obtained with a purity of 78.6%. The purity of DOP was further increased to 95.4% after sevag deproteinization, activated carbon purification, and alcohol precipitation, with a yield of 15.5%, suggesting that polysaccharide was the major component in the DOP and this sample was ready for the in vivo experiment. The monosaccharide composition analysis of DOP with the PMP-HPLC method was then conducted. A comparison of the chromatogram of the standard revealed the monosaccharide composition of DOP was Man and Glc (Figure 1), and the ratio of the monosaccharides was calculated to be Man: Glc = 3.45:1 based on the standard curves of monosaccharides (Table S1). These data indicated that mannose was the main monosaccharide component in DOP, which was consistent with the previous study [32].

### 3.2. Effects of DOP on RRRD Value, Glucose Metabolism, and Lipid Profile of Prediabetic Mice

**Figure 2:**
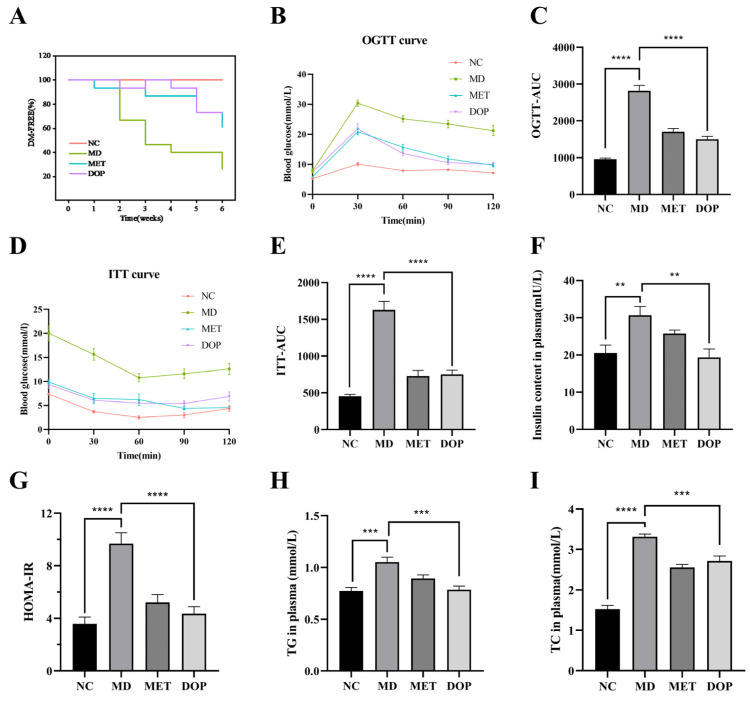
Effects of DOP on RRRD values, glucose tolerance, and lipid metabolism profile of prediabetic mice. The changes in the percentage of diabetes-free mice (%) (RRRD values) after drug administration (**A**). OGTT assay at the 6th week after drug administration, and areas under the OGTT curve (**B**,**C**). ITT assay at the 6th week after drug administration, and areas under the ITT curve (**D**,**E**). The levels of fasting plasma insulin (**F**). The levels of HOMA-IR index (**G**). The levels of TG in plasma (**H**). The levels of TC in plasma (**I**). Values are shown as the mean ± SEM. Comparisons between groups were analyzed using one-way ANOVA followed by Tukey’s post-hoc test. A value of *p* < 0.05 was considered statistically significant, and ** *p* < 0.01, *** *p* < 0.001, **** *p* < 0.0001 vs. mice in the MD group.

The effect of DOP on the prediabetic mice was evaluated after 6 weeks of DOP treatment. The percentages of the prediabetic mice which developed into T2DM mice based on PBG were measured in each group for each week (Figure 2A). The results showed that 73.6% of the mice in the MD group developed into T2DM in Week 6, which was consistent with the risk of prediabetes developing into T2DM in the previous study [8], and the RRRD of the MET group, the positive control, was calculated as 47.1%, which was also consistent with clinical trials [33]. These data indicated that the prediabetes model was established successfully and can be used to evaluate the effect of DOP. The RRRD value in the DOP treatment group was calculated as 63.7%, demonstrating that DOP showed a better effect than metformin in reducing the risk of developing T2DM from prediabetes. 

Meanwhile, the results of OGTT and AUC suggest that DOP exhibited a significant effect on the improvement of glucose tolerance (Figure 2B,C). A significantly increased level of blood glucose was identified in prediabetic mice during the ITT, indicating compensation for the decreased insulin sensitivity (Figure 2D,E). Consistently, the increase in the HOMA-IR index was identified in prediabetic mice (Figure 2G). DOP treatment decreased the level of blood glucose in the ITT test and HOMA-IR index, which was similar to the metformin group (Figure 2D,E,G). These results indicate that DOP alleviated impaired glucose tolerance and insulin resistance in prediabetic mice and showed a positive effect on maintaining stable blood glucose levels and regulating glucose metabolism.

The effects of DOP on lipid metabolism in prediabetes were also investigated. The results showed that HFHSD could lead to increased plasma levels of TG, and TC in prediabetic mice (Figure 2H,I). Research indicated that an increment of TG and TC could cause fat accumulation, causing obesity, and inducing insulin resistance in the body; consequently, leading to elevated blood glucose and the development of diabetes [34]. In our study, TC and TG levels improved significantly after the intervention with DOP (Figure 2H,I), suggesting DOP treatment could regulate lipid metabolism in prediabetes. 

Furthermore, it was found that the levels of AST in the MD group were significantly increased, indicating impaired liver function. The level of AST in the experimental group significantly decreased after DOP intervention. However, another liver enzyme ALT was not influenced by DOP treatment (Figure S1A,B).

### 3.3. Effects of DOP on the Damage of Tissues In Vivo 

**Figure 3:**
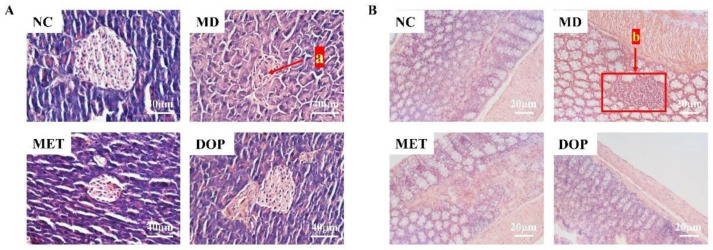
DOP treatment ameliorated the histological damage of the pancreas and colon tissues in prediabetic mice. H&E staining for the evaluation of histological changes in mice pancreas (**A**). H&E staining for the evaluation of histological changes in mice colon (**B**). Tissue sections were observed and photographed at 200× and 100× using a light microscope. Islet damage is indicated by arrow “a”, and inflammatory infiltrates are indicated by arrow “b”.

After H&E staining, the histological analyses of inlets in the pancreas, colon, and liver in each experimental group were performed. The results indicated that the islets in the pancreas of the NC group were intact in shape (Figure 3A), in which the cells in the islets were visible, while the islets of the MD group were damaged to some extent and the number of cells in the islets was reduced (indicated by arrow a). In contrast, islet damage was alleviated in mice that underwent DOP intervention. For the colon, severe inflammatory infiltration was found in the MD group (indicated by arrow b) but was alleviated by the DOP intervention effectively (Figure 3B). For the liver, H&E staining data showed significant cellular steatosis and cell swelling in the MD group compared to the NC group. However, the steatosis was alleviated after the DOP intervention (Figure S2). These data indicated that DOP treatment alleviated the tissue damage caused by prediabetes, which is positive for improving glucose and lipid metabolism in prediabetes.

### 3.4. Effects of DOP on the Gut Microbiota In Vivo and In Vitro

**Figure 4:**
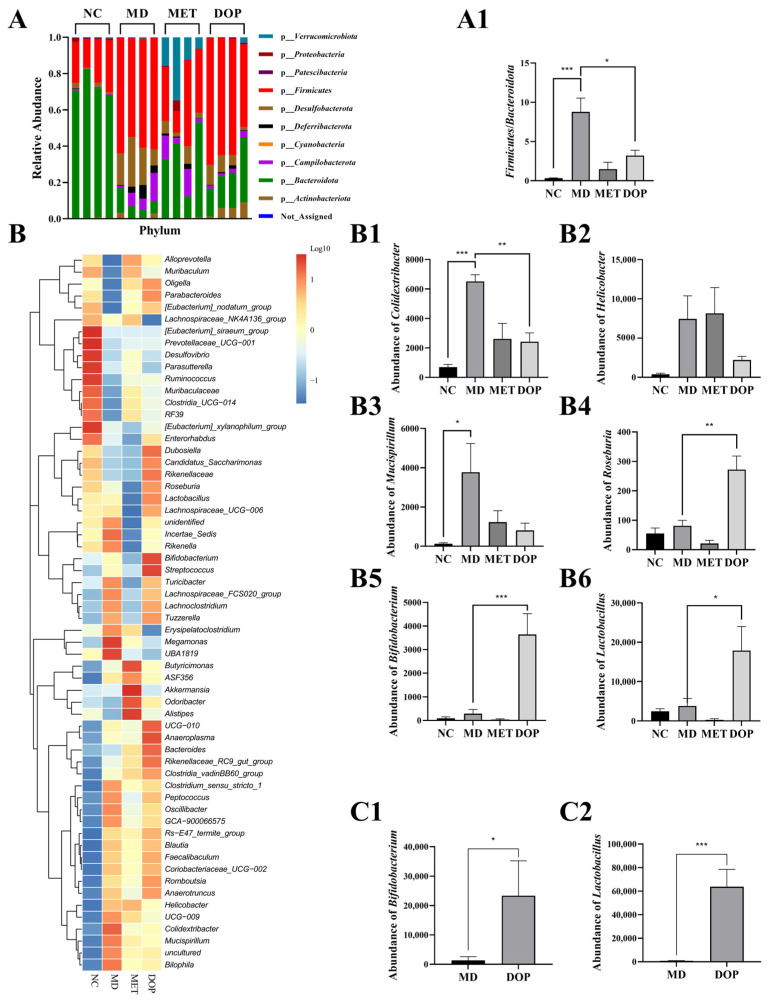
Effects of DOP on the structure of gut microbiota in vivo and in vitro. Taxonomic analysis of gut bacteria in different groups of mice at the phylum level in vivo (**A**). The ratio of *Firmicutes* to *Bacteroidetes* in vivo (**A1**). Genus level clustering heatmap (**B**). Relative abundance of genus *Colidextribacter* (**B1**), *Helicobacter* (**B2**), *Mucispirillum* (**B3**), *Roseburia* (**B4**), *Bifidobacterium* (**B5**), and *Lactobacillus* (**B6**) in vivo. Relative abundance of *Bifidobacterium* (**C1**), and *Lactobacillus* (**C2**) in vitro. Values are shown as the mean ± SEM. Comparisons between groups were analyzed using one-way ANOVA followed by Tukey’s post-hoc test. A value of *p* < 0.05 was considered statistically significant, and * *p* < 0.05, ** *p* < 0.01, *** *p* < 0.001, vs. mice in the MD group.

The feces of each group of mice were also sampled for 16S rRNA sequencing. In terms of the phylum level, an increase in the abundance of *Firmicutes* and a decrease in the abundance of *Bacteroides* was found in the MD group compared to the NC group. This phenomenon was partly reversed by the DOP intervention; the ratio of *Firmicutes*/*Bacteroides* (F/B) was reduced significantly by upregulating the abundance of *Bacteroidota* without obviously influencing the abundance of *Firmicutes* (Figure 4(A1)).

At the level of genus, the abundance of *Akkermansia* was enriched in the MET group (Figure S3A), which was consistent with previous research [35]. The abundance of *Colidextribacter*, *Helicobacter*, and *Mucispirillum*, which were reported as pathogens, were enriched in the MD group, while the abundance of these genera was downregulated by 62.81%, 70.35%, and 78.73% in the DOP group, respectively (Figure 4(B1–B3)). On the contrary, the abundance of some genera, *Roseburia*, *Bifidobacterium*, and *Lactobacillus* (Figure 4(B4–B6)) and *Alloprevotella* and *Bacteroides* (Figure S3B,C) were enriched in the DOP group, particularly *Bifidobacterium* and *Lactobacillus*. The abundances of these genera were 12.65-fold and 4.68-fold higher than that in the MD group, and 40.49-fold, and 7.19-fold higher than that in the NC group, respectively (Figure 4(B5,B6)). 

To exclude the chance that the abundance changes in these genera were caused by other factors, the DOP culture with the gut microbiota in vitro for 12 h using our published method and the abundance of these genera were analyzed by 16S rRNA sequencing [31]. The results showed that DOP significantly upregulated *Bifidobacterium* and *Lactobacillus* abundance in vitro (Figure 4(C1,C2)). 

### 3.5. DOP Improved Insulin Resistance through Regulation of TLR4 Pathway In Vivo

**Figure 5:**
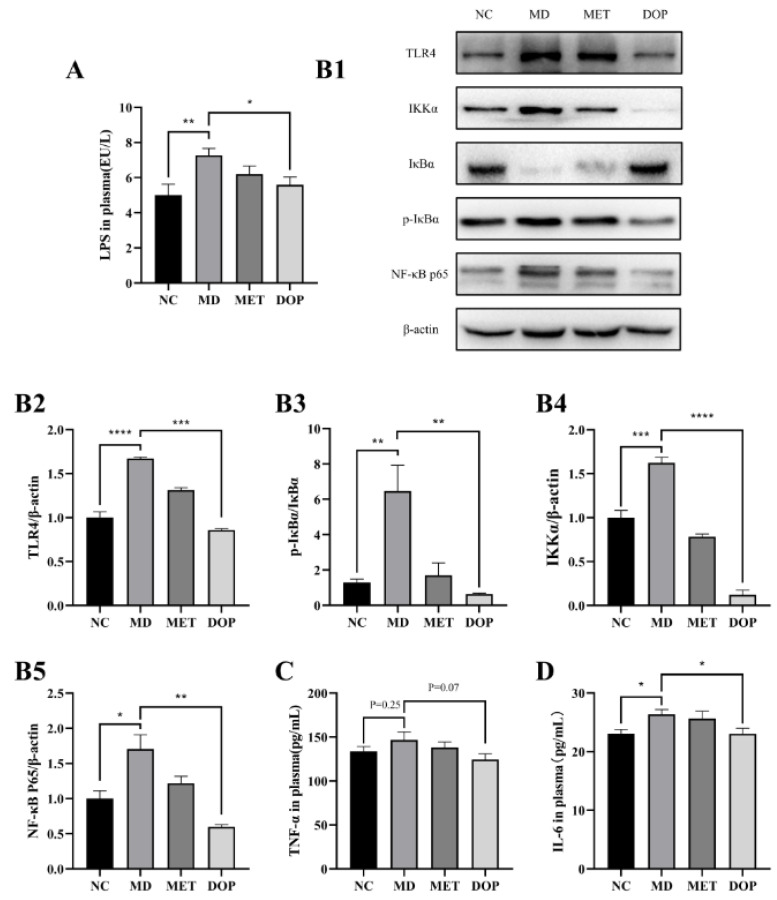
Effects of DOP treatment on TLR4/NF-κB signaling pathway. Effect of DOP on plasma LPS level (**A**). The Western blotting results of TLR4, IKKβ, IκBα, p-IκBα, and NF-κB p65 in different groups (**B1**). Grayscale analysis of TLR4 (**B2**), IKKβ (**B3**), p-IκBα/IκBα (**B4**), and NF-κB p65 (**B5**), which were normalized by β-actin. Effect of DOP on plasma TNF-α and IL-6 level (**C**,**D**). Values are shown as the mean ± SEM. Comparisons between groups were analyzed using one-way ANOVA followed by Tukey’s post-hoc test. A value of *p* < 0.05 was considered statistically significant, and * *p* < 0.05, ** *p* < 0.01, *** *p* < 0.001, **** *p* < 0.0001 vs. mice in the MD group.

Our data indicated that DOP improved insulin resistance in prediabetic mice. Dysbiosis of the gut microbiota leads to inflammation and dysfunction in the body [36], and systemic inflammation has been shown to be strongly associated with insulin resistance and the occurrence of T2DM [37]. Thus, in this study, the effects of DOP on systemic inflammation were also investigated. The MD group showed a significant increase in plasma LPS levels, as well as the inflammatory factors, TNF-α, and IL-6, compared to the NC group, and DOP had an ameliorating effect on LPS, TNF-α, and IL-6 levels (Figure 5A,C,D). To understand the mechanism of the anti-inflammation of DOP, Western blotting analyses of colonic tissue were performed, and we found that the expressions of TLR4, IKKα, p-IκBα/IκBα, and NF-κB p65 were significantly elevated in the MD group compared with the NC group and decreased in the DOP treatment group (Figure 5(B1–B5)). The results above demonstrated that DOP improved insulin resistance in prediabetic mice by ameliorating systemic inflammation. 

### 3.6. DOP Alleviated the Pancreatic Islet Damage, Reduced Water and Diet Intake, and Improved Insulin Resistance through the Regulation of the SCFA-FFAR2/FFAR3 Pathway In Vivo

**Figure 6:**
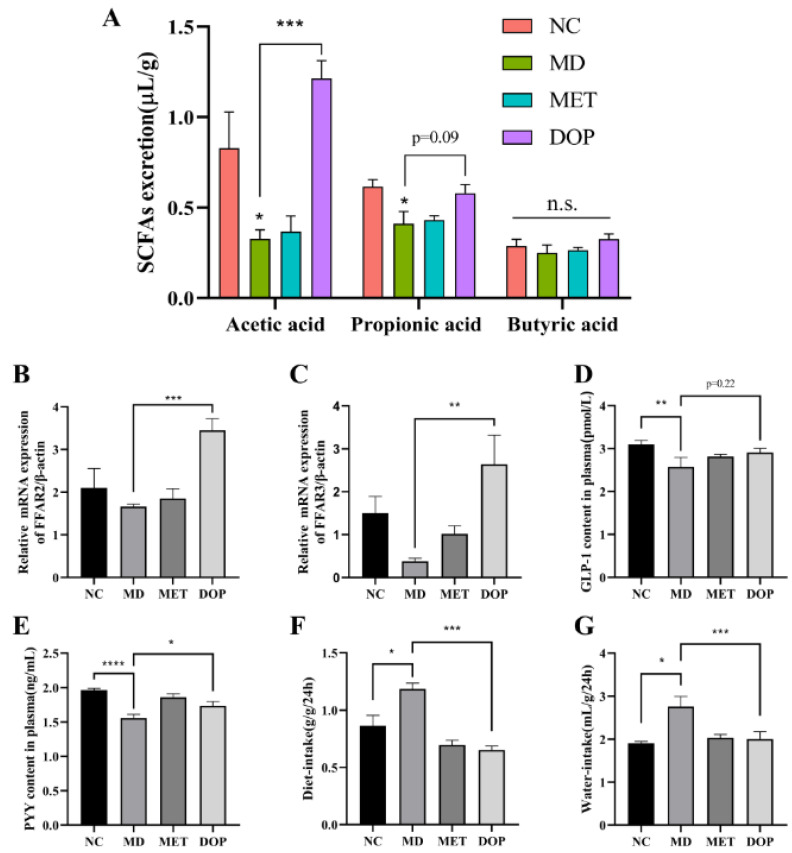
Effect of DOP treatment on SCFA-FFAR2/FFAR3 pathway. Effect of DOP on intestinal SCFA levels (**A**). The RT-PCR results of FFAR2 and FFAR3 in different groups (**B**,**C**), which was normalized by β-actin. Effects of DOP on plasma GLP-1 and PYY levels (**D**,**E**). Effects of DOP on diet intake and water intake (**F**,**G**). Values are shown as the mean ± SEM. Comparisons between groups were analyzed using one-way ANOVA followed by Tukey’s post-hoc test. A value of *p* < 0.05 was considered statistically significant, and * *p* < 0.05, ** *p* < 0.01, *** *p* < 0.001, **** *p* < 0.0001 vs. mice in the MD group.

As major metabolites of gut microbiota, SCFAs were regarded to play an important role in hindering DM development [36,38]. Thus, the levels of three kinds of SCFAs in each group were measured and compared (Figure 6A). The concentrations of acetic acid, propionic acid, and butyric acid in the MD group were significantly decreased compared with those in the NC group, the administration of DOP significantly increased the levels of total SCFAs and acetic acid, probably due to the regulation of the abundance of SCFA-producing bacteria. For propionic acid and butyric acid, the administration of DOP increased the levels of these two SCFAs but without significance.

Free fatty acid receptors FFAR2/FFAR3 are important receptors for SCFAs and are widely expressed in adipose tissue, the intestinal tract, and the peripheral nervous system [37]. Recent research indicated that FFAR2/FFAR3 stimulated the expression of the intestinal hormone GLP-1 and PYY, and consequently influenced glucose metabolism in diabetic patients [37,38,39]. The relationship between FFAR2/FFAR3 with insulin resistance was also well documented [40,41]. Thus, the FFAR2/FFAR3 transcriptional activities were assayed in intestinal tissues and the levels of their downstream signal factors were also measured. The results indicated that DOP increased the FFAR2/FFAR3 expression significantly (Figure 6B,C). Meanwhile, the plasma levels of GLP-1 and PYY in the MD group were significantly lower than those in the NC group (Figure 6D,E), and metformin intervention cannot increase the level of GLP-1. However, higher GLP-1 and PYY levels were observed in the DOP treatment group than those in the MD group. These data suggested that administration of DOP increased the abundance of SCFA-producing bacteria, which led to the increment of SCFA levels, especially the acetic acid level, and consequently enhanced the expression of the free fatty acid receptors FFAR2/FFAR3, which then increased the expression of the intestinal hormone GLP-1 and PYY to improve the glucose metabolism. To confirm this speculation, the water and diet intake of each group, which was reported to be closely related to GLP-1 and PYY [42], was measured and is shown in Figure 6F,G. As expected, significantly higher water and diet intake was observed in the MD group compared to those of the NC group, and the DOP administration reduced the water and diet intake significantly. In addition, the upregulation of FFAR2, FFAR3, GLP-1, and PYY was reported to improve insulin resistance [36]. The elevation of FFAR2, FFAR3, GLP-1, and PYY by DOP treatment was another mechanism of insulin resistance improvement in prediabetic mice.

## 4. Discussion

The incidence of diabetes is increasing annually, and this is closely linked to the high incidence of prediabetes. Therefore, the search for effective means that can prevent the progression from prediabetes to T2DM is of great importance. Most of the currently available hypoglycemic drugs have side effects; therefore, the active ingredients in natural plants are gradually becoming a research hotspot because of their unique hypoglycemic activity and low side effects. *D. officinale* has been under public attention for thousands of years, and the polysaccharide, as the main component, has been reported in many kinds of literature for its hypoglycemic activity. However, studies on the modulatory effect of DOP on prediabetes have not been reported. Thus, our data in this study provided an important basis for the exploitation of DOP as a novel functional food additive in the prevention of T2DM. 

Numerous studies have found that the administration of polysaccharides is associated with a change in the gut microbiota of diabetic animals [43]. However, for DOPs, only a few studies were reported with ameliorative effects on T2DM, which were associated with significant changes in the structure of the gut microbiota. In this study, the structure of the gut microbiota of prediabetic mice altered by DOP treatment was reported as significant for the first time. Previous research has indicated that the F/B ratio is closely associated with inflammation, insulin resistance, and elevated blood glucose level, which influenced the development of T2DM [44,45]; thus, the downregulation of the F/B ratio by DOP may play a positive impact on the glucose metabolism of prediabetes. The previous study indicated that *Alloprevotella*, *Bifidobacterium*, *Bacteroides*, *Lactobacillus*, and *Roseburia*, which were also observed to increase in this study, were negatively associated with T2DM by alleviating inflammation or regulating glucose/insulin homeostasis [44]. Thus, elevating the abundance of these genera might contribute to inhibiting the progression of T2DM. It is necessary to point out that previously the composition change in gut microbiota regulated by DOP was mainly investigated by in vivo models, as the multi-target effects of DOP in vivo, and whether the abundance change in these genera in the gut microbiota was induced by DOP directly, still lacks evidence. In this study, using the in vitro gut microbiota model developed by our group [31], the obvious increment of *Bifidobacterium* and *Lactobacillus* abundance was detected, which is, to our best knowledge, the first report showing that DOP could increase the abundance of *Bifidobacterium* and *Lactobacillus* in the gut microbiota directly, thus providing a theoretical basis for the application of DOP as a regulator of gut microbiota.

Research indicated that SCFAs are produced by the gut microbiota by breaking down dietary fiber, undigested starch, and carbohydrates that are not absorbed by the gastrointestinal tract. Keeping the content of SCFAs at the proper level is beneficial to the normal physiological state; however, in some diseases, such as diabetes or prediabetes, the levels of SCFA in the intestinal tract were significantly reduced [39]. Non-starchy natural polysaccharide is not readily broken down by the enzymes produced by the body and will mostly enter the large intestine to intervene in the production of SCFAs by upregulating the abundance of SCFA-producing bacteria, including *Alloprevotella*, *Bifidobacterium*, *Bacteroides*, *Lactobacillus*, and *Roseburia*, and the biological activity of the enzymes that catalyze the production of SCFAs [45,46], and a similar result was observed for DOP in our study. Therefore, DOP was supposed to increase the content of SCFAs by upregulating the abundance of SCFA-producing bacteria. This phenomenon was also reported by a previous study [20]; however, the linkage between the hypoglycemic activity exerted by DOP and the changes in SCFA levels were still missing. As a step forward, we investigated the effect of DOP on SCFA receptors, and the subsequent elevated production of intestinal hormones, elucidating the detailed mechanism for the beneficial effect of DOP via SCFAs.

Several previous types of research suggested that SCFAs bind to intestinal L-cell short-chain fatty acid receptors FFAR2/FFAR3 and stimulate the secretion of the intestinal hormones GLP-1 and PYY by colonic cells [39,47,48]. GLP-1 alleviated the apoptosis of islet cells to increase insulin secretion and insulin sensitivity [49], while PYY was negatively associated with excessive food intake and IR [50]. Our data demonstrated that DOP treatment led to the upregulated expression of FFAR2/FFAR3, increasing the levels of GLP-1 and PYY in the plasma. In this study, the simulation of PYY secretion by DOP was reported for the first time, which will help to understand the relationship between the DOP administration and food intake. The simulation of GLP-1 secretion in diabetic mice by DOP was reported by Hu et al., and GLP-1 production was demonstrated to be involved in Ca^2+^/CAM/CaMKII and MAPK pathways [26]. However, due to the large molecular weight of DOP and the fact that both the small intestine and colon are covered with a mucus layer to form the intestinal barrier, which blocks the entry of biomacromolecules into intestinal cells [51], in our opinion, the effects of DOP on the intestinal microenvironment might also play an important role in the regulation of GLP-1 secretion by DOP. Therefore, to the best of our knowledge, this is the first report revealing the mechanism that the structural and abundance changes in gut microbiota caused by DOP intervention reduced the intake of food and water and alleviated the damage of islet cells in prediabetic or diabetic mice.

Some typical pathogens, including the genus of *Colidextribacter*, *Mucispirillum*, and *Helicobacter*, were also reported to be positively associated with inflammation [52,53,54]. Increasing the abundance of these pathogens can affect immune cells and consequently induce inflammatory reactions in the gut directly and indirectly through microbial products, including LPS, metabolites, and SCFAs [55], all of which can eventually affect insulin resistance [56]. LPS was reported to bind to TLR4 and consequently activate a wide range of inflammatory pathways, in particular the NF-κB pathway [57]. In addition, SCFAs activated FFAR2/FFAR3 and increased the interaction between β-arrestin-2 and I-κBα, and then inhibited NF-κB expression [37,58]. The regulation of the TLR4 pathway by DOP treatment has been reported in vitro [59,60]; however, the mechanism on the regulation of the TLR4 signal pathway by DOP to improve insulin resistance was not documented. In this study, the abundance of some inflammation-related pathogens was reduced significantly, and DOP was found to reduce inflammation in prediabetic mice through its ameliorative effect on the LPS/TLR4 signaling pathway and the upregulation of the FFAR2/FFAR3 signaling pathway. Therefore, our study demonstrated the mechanism of DOP on the improvement of insulin sensitivity through altering the gut microbiota and regulation of inflammation-related pathways in prediabetic mice.

**Figure 7:**
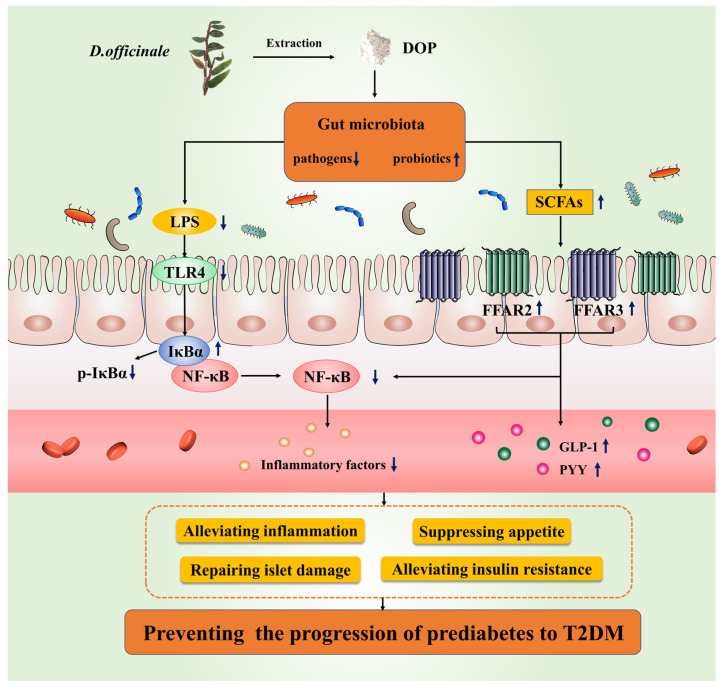
The mechanism of DOP preventing T2DM in prediabetic mice.

Glucomannan is one of the most important bioactive polysaccharides, and it consists of the linear chain of mixed residues of 1, 4 linked D-mannose and D-glucose monomers arranged in blocks [61]. Natural glucomannans have been found to exhibit a variety of biological activities, including hypoglycemic activity, despite differences in the structure of these glucomannans [61,62]. For example, konjac glucomannan exhibited anti-diabetic activity by delaying glucose uptake or regulating the insulin signaling pathway [63,64]. These data suggested that glucomannan lowered blood glucose levels in diabetes through various mechanisms. The regulation effects of glucomannan on gut microbiota were also reported [65]; however, to the best of our knowledge, there is still a lack of a detailed mechanism that linked gut microbiota regulation with hypoglycemic activity. In this study, the detailed hypoglycemic mechanism of the DOP, a glucomannan, in preventing the progression of prediabetes to T2DM via the regulation of gut microbiota was revealed (Figure 7). Our work will lay a solid basis for the application of DOP in the field of diabetes prevention. Admittedly, there are still some deficiencies in this work; for example, only a single dosage of DOP was used in the animal experiment, and the type of glycosidic bond in purified DOP is still unclear, which needs to be investigated in the future.

## Figures and Tables

**Table 1:** Primer sequences of FFAR2 and FFAR3.

Genes		Primer (5′-3′)
FFAR2	forward	GGACCAGAGGAGAACCAGGTAGAAG
	reverse	GCCGTGAGGATCAAGGAACTGTG
FFAR3	forward	CCACACTGCTCATCTTCTTCGTCTG
	reverse	ACGGACTCTCACGGCTGACATAG

## Data Availability

The data presented in this study are available in the Supplementary Materials.

## Supplementary Materials

The following supporting information can be downloaded at: https://www.mdpi.com/article/10.3390/foods12122310/s1, Table S1: Monosaccharide standard curve of PMP-HPLC; Figure S1: Effect of DOP or metformin on the level of ALT and AST; Figure S2: H&E staining for the evaluation of histological changes in mice liver and Figure S3: Relative abundance of *Akkermansia*, *Alloprevotella*, and *Bacteroides* in vivo.
