# Supplementary material for: Dendrobium officinale Polysaccharide Prevents Diabetes via the Regulation of Gut Microbiota in Prediabetic Mice
Source: Foods. 2023 Jun 8;12(12):2310. doi: 10.3390/foods12122310 (PMC10296996; doi:10.3390/foods12122310)
Supplement: Supplementary file 1 [file foods-12-02310-s001.zip › foods-2412040-supplementary.pdf]

Supplmention

Table S1 Monosaccharide standard curve of PMP-HPLC

Type of monosaccharide	standard curve	R^2
Man	y = 13420894.70 x -111874.07	0.9999
Glc	y = 8403312.60 x + + 349394.33	0.9986

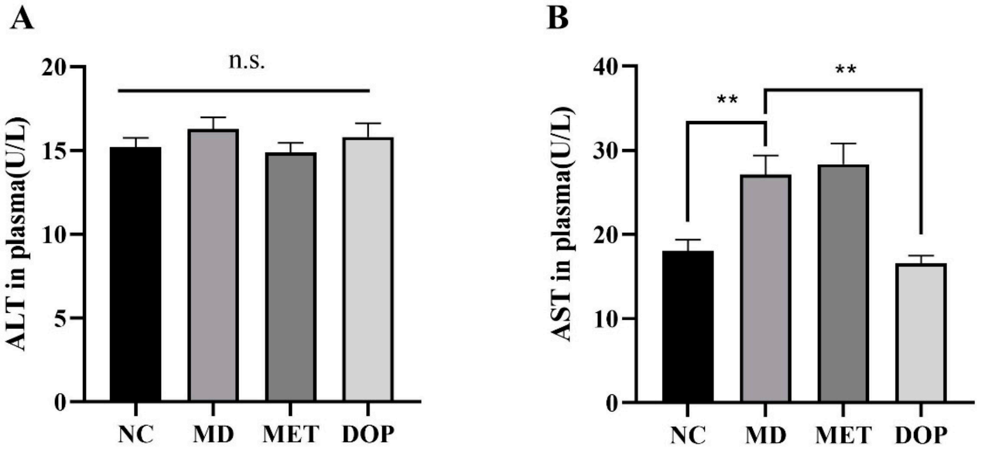

Figure S1. (A,B) Effect of DOP or metformin on the level of ALT and AST. Values are shown as the mean ± SEM. Comparisons between groups were analyzed using one-way ANOVA followed by Tukey's post-hoc test. A value of $P < 0.05$ was considered statistically significant, and **$p < 0.01$ vs. mice in the MD group.

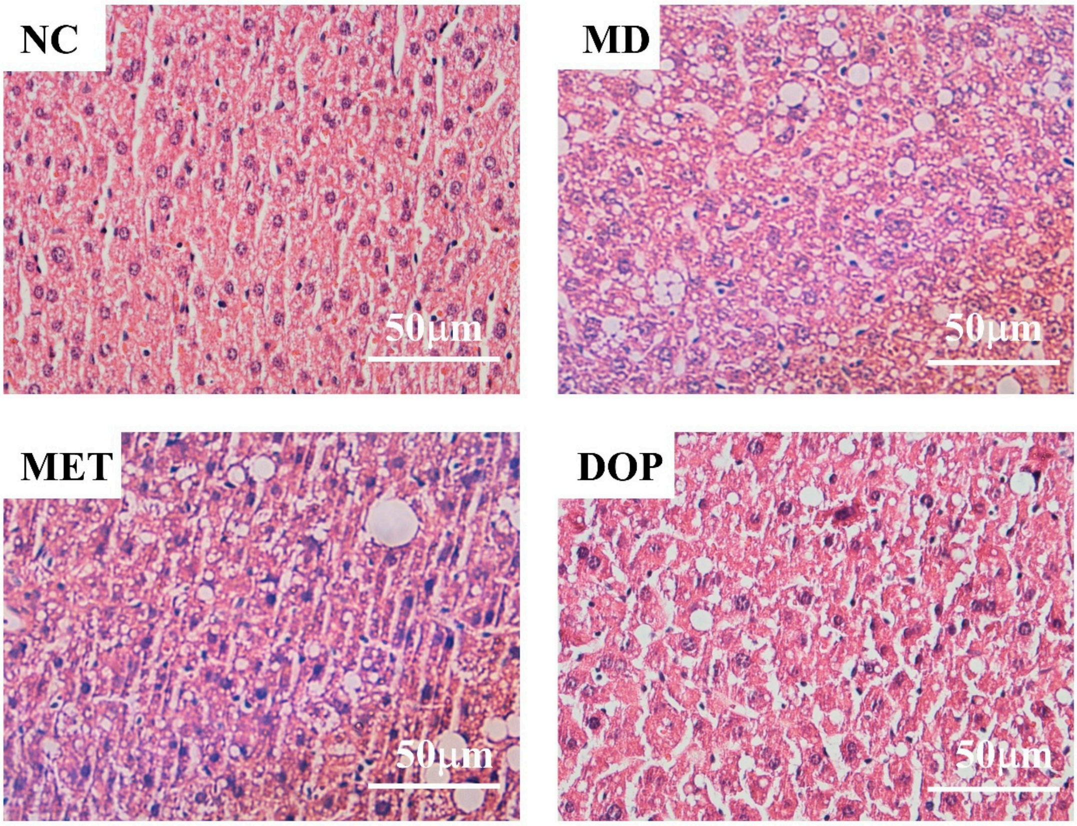

Figure S2. H&E staining for the evaluation of histological changes in mice liver. Tissue sections were observed and photographed at 200× using a light microscope.

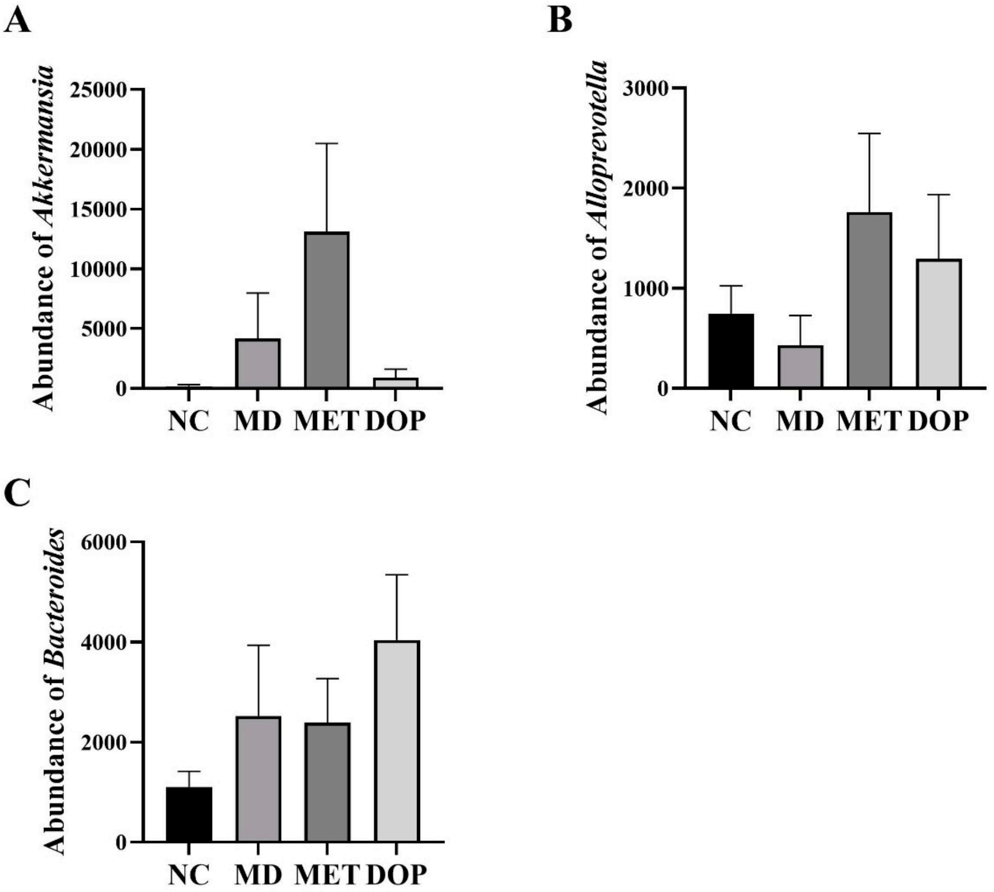

Figure S3. (**A**,**B**,**C**) **Relative abundance of *Akkermansia*, *Alloprevotella*, and *Bacteroides* in *vivo*.** Values are shown as the mean ± SEM. Comparisons between groups were analyzed using one-way ANOVA followed by Tukey's post-hoc test. A value of $P < 0.05$ was considered statistically significant.
